# Supplementary material for: Hot spot prediction in protein-protein interactions by an ensemble system
Source: BMC Syst Biol. 2018 Dec 31;12(Suppl 9):132. doi: 10.1186/s12918-018-0665-8 (PMC6311905; doi:10.1186/s12918-018-0665-8)
Supplement: Supplementary file 2 — All relevant MATLAB code. A simple Matlab implement of our predictor. (ZIP 19,742 kb) [file 12918_2018_665_MOESM2_ESM.zip › Additional file 2/Read ASA/ASA website/dbMPIKT/4Y61B.html]

 NetSurfP - Protein Surface Accessibility and Secondary Structure Predictions

  

|  |  |  |
| --- | --- | --- |
|  |  | NetSurfP - Protein Surface Accessibility and Secondary Structure Predictions  Technical University of Denmark |

  


---

```
# For publication of results, please cite:
# A generic method for assignment of reliability scores applied to solvent accessibility predictions.
# Bent Petersen, Thomas Nordahl Petersen, Pernille Andersen, Morten Nielsen and Claus Lundegaard
# BMC Structural Biology 2009, 9:51 doi:10.1186/1472-6807-9-51
#
# Column 1: Class assignment - B for buried or E for Exposed - Threshold: 25% exposure, but not based on RSA
# Column 2: Amino acid
# Column 3: Sequence name
# Column 4: Amino acid number
# Column 5: Relative Surface Accessibility - RSA
# Column 6: Absolute Surface Accessibility
# Column 7: Z-fit score for RSA prediction
# Column 8: Probability for Alpha-Helix
# Column 9: Probability for Beta-strand
# Column 10: Probability for Coil
E M  4Y61B                  1    0.695 139.009  -2.109   0.003   0.003   0.994
B L  4Y61B                  2    0.245  44.860  -1.062   0.257   0.016   0.727
E S  4Y61B                  3    0.393  46.071  -2.351   0.522   0.016   0.462
B G  4Y61B                  4    0.315  24.790  -1.939   0.802   0.014   0.185
B V  4Y61B                  5    0.252  38.671  -1.209   0.879   0.010   0.111
B W  4Y61B                  6    0.206  49.615   0.860   0.938   0.007   0.055
B F  4Y61B                  7    0.220  44.094  -0.255   0.970   0.001   0.030
B L  4Y61B                  8    0.199  36.474   0.676   0.970   0.001   0.030
B S  4Y61B                  9    0.234  27.472   0.373   0.970   0.001   0.030
B V  4Y61B                 10    0.179  27.528   0.913   0.970   0.001   0.030
B L  4Y61B                 11    0.211  38.671   0.625   0.970   0.001   0.030
B T  4Y61B                 12    0.193  26.769  -0.278   0.970   0.001   0.030
B V  4Y61B                 13    0.154  23.654  -0.035   0.923   0.002   0.076
B A  4Y61B                 14    0.192  21.147  -1.250   0.923   0.002   0.076
B G  4Y61B                 15    0.185  14.575  -0.509   0.923   0.002   0.076
B I  4Y61B                 16    0.113  20.979  -0.458   0.858   0.002   0.139
B L  4Y61B                 17    0.102  18.749  -0.187   0.782   0.003   0.216
B Q  4Y61B                 18    0.170  30.380  -0.512   0.694   0.003   0.303
B T  4Y61B                 19    0.137  19.002  -1.748   0.600   0.003   0.397
B E  4Y61B                 20    0.273  47.693  -1.118   0.406   0.004   0.590
B S  4Y61B                 21    0.273  31.996  -1.409   0.246   0.004   0.750
E R  4Y61B                 22    0.401  91.783  -1.023   0.176   0.004   0.820
E K  4Y61B                 23    0.415  85.365  -1.279   0.053   0.005   0.942
E T  4Y61B                 24    0.510  70.751  -2.066   0.058   0.017   0.925
E A  4Y61B                 25    0.409  45.028  -1.459   0.018   0.019   0.964
E K  4Y61B                 26    0.527 108.342  -1.140   0.018   0.019   0.964
E D  4Y61B                 27    0.582  83.895  -0.718   0.018   0.019   0.964
E I  4Y61B                 28    0.367  67.803  -0.046   0.005   0.015   0.979
B C  4Y61B                 29    0.043   5.981  -0.475   0.005   0.015   0.979
B K  4Y61B                 30    0.172  35.360  -0.455   0.005   0.015   0.979
E I  4Y61B                 31    0.596 110.334  -0.899   0.005   0.015   0.979
E R  4Y61B                 32    0.526 120.431   0.796   0.004   0.085   0.910
B C  4Y61B                 33    0.034   4.731  -1.180   0.005   0.262   0.733
E L  4Y61B                 34    0.445  81.425   0.832   0.004   0.616   0.381
B C  4Y61B                 35    0.044   6.178  -0.844   0.004   0.514   0.481
E E  4Y61B                 36    0.589 102.898   1.103   0.004   0.197   0.799
E E  4Y61B                 37    0.668 116.769  -0.589   0.005   0.045   0.951
E K  4Y61B                 38    0.324  66.626   0.200   0.018   0.019   0.964
E E  4Y61B                 39    0.695 121.416  -0.638   0.018   0.019   0.964
E N  4Y61B                 40    0.558  81.633   0.867   0.018   0.047   0.935
B V  4Y61B                 41    0.184  28.219   0.383   0.005   0.336   0.660
E L  4Y61B                 42    0.384  70.384   1.057   0.004   0.616   0.381
E N  4Y61B                 43    0.364  53.246   1.534   0.001   0.900   0.099
B I  4Y61B                 44    0.030   5.494   0.323   0.001   0.959   0.040
E N  4Y61B                 45    0.295  43.144   1.180   0.001   0.900   0.099
B C  4Y61B                 46    0.030   4.184  -0.188   0.004   0.616   0.381
E E  4Y61B                 47    0.403  70.317   0.358   0.005   0.262   0.733
E N  4Y61B                 48    0.654  95.687  -0.464   0.005   0.045   0.951
E K  4Y61B                 49    0.451  92.750   0.551   0.018   0.019   0.964
E G  4Y61B                 50    0.557  43.852   0.642   0.018   0.019   0.964
B F  4Y61B                 51    0.064  12.785  -0.205   0.052   0.084   0.864
E T  4Y61B                 52    0.472  65.425   1.180   0.021   0.279   0.699
E T  4Y61B                 53    0.533  73.872   1.399   0.064   0.216   0.721
B V  4Y61B                 54    0.092  14.110   1.305   0.052   0.084   0.864
B S  4Y61B                 55    0.170  19.912   0.415   0.058   0.017   0.925
E L  4Y61B                 56    0.696 127.419   0.836   0.181   0.016   0.803
E L  4Y61B                 57    0.604 110.629  -0.671   0.115   0.016   0.868
B Q  4Y61B                 58    0.091  16.253   0.333   0.058   0.017   0.925
E P  4Y61B                 59    0.446  63.273  -0.914   0.058   0.017   0.925
E P  4Y61B                 60    0.611  86.687  -0.654   0.058   0.017   0.925
B Q  4Y61B                 61    0.267  47.740  -0.719   0.058   0.017   0.925
E Y  4Y61B                 62    0.407  86.912  -0.450   0.018   0.088   0.893
E R  4Y61B                 63    0.465 106.554   1.002   0.020   0.205   0.775
B I  4Y61B                 64    0.075  13.801   0.205   0.022   0.359   0.619
E Y  4Y61B                 65    0.321  68.705   0.865   0.023   0.655   0.322
E Q  4Y61B                 66    0.392  70.065   1.362   0.011   0.918   0.071
B L  4Y61B                 67    0.040   7.379   0.267   0.011   0.918   0.071
E F  4Y61B                 68    0.297  59.628   0.908   0.018   0.846   0.136
B L  4Y61B                 69    0.054   9.924  -0.150   0.021   0.279   0.699
E N  4Y61B                 70    0.452  66.114  -0.074   0.018   0.088   0.893
E G  4Y61B                 71    0.565  44.473  -0.068   0.018   0.047   0.935
B N  4Y61B                 72    0.119  17.422  -0.722   0.018   0.088   0.893
E L  4Y61B                 73    0.426  77.964   1.199   0.021   0.279   0.699
B L  4Y61B                 74    0.051   9.320  -0.250   0.021   0.451   0.528
E T  4Y61B                 75    0.429  59.572   1.249   0.021   0.451   0.528
E R  4Y61B                 76    0.527 120.591   1.539   0.022   0.359   0.619
B L  4Y61B                 77    0.062  11.407   0.987   0.020   0.205   0.775
E Y  4Y61B                 78    0.406  86.677   0.922   0.018   0.047   0.935
E P  4Y61B                 79    0.620  88.035   0.436   0.406   0.004   0.590
E N  4Y61B                 80    0.613  89.772  -0.086   0.502   0.002   0.495
B E  4Y61B                 81    0.190  33.106  -0.002   0.502   0.002   0.495
B F  4Y61B                 82    0.070  13.969  -1.935   0.522   0.016   0.462
E V  4Y61B                 83    0.559  85.964   0.840   0.321   0.003   0.675
E N  4Y61B                 84    0.573  83.873   0.591   0.176   0.004   0.820
B Y  4Y61B                 85    0.071  15.173   0.683   0.058   0.017   0.925
E S  4Y61B                 86    0.534  62.585   0.745   0.053   0.043   0.903
E N  4Y61B                 87    0.427  62.542   1.557   0.052   0.084   0.864
B A  4Y61B                 88    0.047   5.234   0.330   0.019   0.141   0.840
E V  4Y61B                 89    0.328  50.414   1.452   0.022   0.359   0.619
E T  4Y61B                 90    0.403  55.924   1.765   0.011   0.918   0.071
B L  4Y61B                 91    0.037   6.683   0.637   0.011   0.918   0.071
E H  4Y61B                 92    0.285  51.751   0.995   0.018   0.846   0.136
B L  4Y61B                 93    0.044   8.038  -0.319   0.022   0.359   0.619
E G  4Y61B                 94    0.444  34.951   0.110   0.018   0.088   0.893
E N  4Y61B                 95    0.612  89.538   0.279   0.005   0.015   0.979
B N  4Y61B                 96    0.118  17.319  -0.496   0.018   0.047   0.935
E G  4Y61B                 97    0.432  33.998   1.485   0.021   0.279   0.699
B L  4Y61B                 98    0.054   9.906  -0.341   0.022   0.552   0.426
E Q  4Y61B                 99    0.456  81.477   1.368   0.004   0.514   0.481
E E  4Y61B                100    0.527  92.154   1.594   0.004   0.514   0.481
B I  4Y61B                101    0.061  11.377   1.383   0.005   0.262   0.733
E R  4Y61B                102    0.406  92.928   1.173   0.018   0.047   0.935
E P  4Y61B                103    0.661  93.796   0.930   0.600   0.003   0.397
E G  4Y61B                104    0.553  43.513   0.326   0.600   0.003   0.397
B A  4Y61B                105    0.104  11.417  -0.278   0.600   0.003   0.397
B F  4Y61B                106    0.098  19.729  -1.605   0.522   0.016   0.462
E S  4Y61B                107    0.582  68.175   1.118   0.406   0.004   0.590
E G  4Y61B                108    0.550  43.269   0.922   0.246   0.004   0.750
B L  4Y61B                109    0.054   9.851   0.520   0.176   0.004   0.820
E K  4Y61B                110    0.562 115.521   1.358   0.257   0.016   0.727
E T  4Y61B                111    0.462  64.066   2.013   0.191   0.086   0.723
B L  4Y61B                112    0.055  10.034   0.888   0.052   0.084   0.864
E K  4Y61B                113    0.368  75.636   1.576   0.064   0.216   0.721
E R  4Y61B                114    0.415  94.966   1.852   0.018   0.846   0.136
B L  4Y61B                115    0.035   6.335   0.695   0.018   0.846   0.136
E H  4Y61B                116    0.296  53.915   0.905   0.021   0.756   0.223
B L  4Y61B                117    0.044   8.111  -0.620   0.021   0.279   0.699
E N  4Y61B                118    0.465  68.061  -0.137   0.005   0.045   0.951
E N  4Y61B                119    0.611  89.436  -0.197   0.018   0.019   0.964
B N  4Y61B                120    0.137  20.071  -0.856   0.018   0.047   0.935
E K  4Y61B                121    0.414  85.119   1.465   0.064   0.216   0.721
B L  4Y61B                122    0.051   9.393  -0.052   0.069   0.386   0.545
E E  4Y61B                123    0.454  79.349   1.215   0.021   0.451   0.528
E V  4Y61B                124    0.450  69.242   1.602   0.022   0.359   0.619
B L  4Y61B                125    0.048   8.844   0.824   0.019   0.141   0.840
E R  4Y61B                126    0.362  82.829   1.312   0.058   0.017   0.925
E E  4Y61B                127    0.645 112.716   0.529   0.600   0.003   0.397
E D  4Y61B                128    0.561  80.768  -0.298   0.600   0.003   0.397
B T  4Y61B                129    0.144  19.959   0.322   0.522   0.016   0.462
B F  4Y61B                130    0.093  18.745  -0.960   0.522   0.016   0.462
E L  4Y61B                131    0.569 104.166   0.771   0.321   0.003   0.675
E G  4Y61B                132    0.534  42.018   0.534   0.176   0.004   0.820
B L  4Y61B                133    0.054   9.814   0.079   0.109   0.005   0.886
E E  4Y61B                134    0.539  94.163   0.974   0.257   0.016   0.727
E S  4Y61B                135    0.412  48.310   1.658   0.184   0.043   0.773
B L  4Y61B                136    0.049   8.990   0.525   0.113   0.043   0.844
E E  4Y61B                137    0.342  59.817   1.255   0.056   0.142   0.802
E Y  4Y61B                138    0.416  89.006   1.889   0.085   0.775   0.140
B L  4Y61B                139    0.042   7.763   0.528   0.072   0.855   0.073
E Q  4Y61B                140    0.309  55.205   0.638   0.085   0.775   0.140
B A  4Y61B                141    0.048   5.334  -0.183   0.020   0.205   0.775
E D  4Y61B                142    0.437  63.029  -0.294   0.018   0.047   0.935
E Y  4Y61B                143    0.599 127.964  -0.209   0.018   0.019   0.964
B N  4Y61B                144    0.101  14.860  -0.842   0.018   0.047   0.935
E Y  4Y61B                145    0.398  85.095   1.299   0.021   0.279   0.699
B I  4Y61B                146    0.056  10.434  -0.584   0.021   0.451   0.528
E S  4Y61B                147    0.380  44.524   1.008   0.004   0.514   0.481
E T  4Y61B                148    0.466  64.690   1.082   0.004   0.514   0.481
B I  4Y61B                149    0.055  10.249   1.203   0.005   0.262   0.733
E E  4Y61B                150    0.319  55.712   1.092   0.018   0.047   0.935
E A  4Y61B                151    0.621  68.401   0.803   0.694   0.003   0.303
E G  4Y61B                152    0.573  45.071   0.111   0.694   0.003   0.303
B A  4Y61B                153    0.101  11.174   0.034   0.600   0.003   0.397
B F  4Y61B                154    0.106  21.354  -1.856   0.600   0.003   0.397
E S  4Y61B                155    0.582  68.257   0.512   0.321   0.003   0.675
E K  4Y61B                156    0.592 121.713   0.443   0.246   0.004   0.750
B L  4Y61B                157    0.068  12.396   0.096   0.053   0.005   0.942
E N  4Y61B                158    0.533  78.046   0.841   0.115   0.016   0.868
E K  4Y61B                159    0.417  85.859   1.764   0.053   0.043   0.903
B L  4Y61B                160    0.051   9.283   0.408   0.052   0.084   0.864
E K  4Y61B                161    0.357  73.414   1.410   0.064   0.216   0.721
E V  4Y61B                162    0.412  63.355   1.959   0.085   0.775   0.140
B L  4Y61B                163    0.038   6.958   0.522   0.018   0.846   0.136
E I  4Y61B                164    0.303  56.074   0.930   0.085   0.775   0.140
B L  4Y61B                165    0.042   7.745  -0.791   0.066   0.296   0.638
E N  4Y61B                166    0.446  65.250   0.059   0.018   0.047   0.935
E D  4Y61B                167    0.595  85.725  -0.252   0.018   0.019   0.964
B N  4Y61B                168    0.112  16.441  -0.738   0.018   0.047   0.935
E L  4Y61B                169    0.411  75.254   1.305   0.064   0.216   0.721
B L  4Y61B                170    0.048   8.844  -0.464   0.066   0.296   0.638
E L  4Y61B                171    0.437  80.070   1.381   0.069   0.386   0.545
E S  4Y61B                172    0.417  48.837   1.162   0.064   0.216   0.721
B L  4Y61B                173    0.057  10.400   1.350   0.191   0.086   0.723
E P  4Y61B                174    0.285  40.470   0.827   0.257   0.016   0.727
E S  4Y61B                175    0.634  74.281   0.656   0.522   0.016   0.462
E N  4Y61B                176    0.537  78.675  -0.766   0.406   0.004   0.590
B V  4Y61B                177    0.113  17.414  -1.267   0.406   0.004   0.590
B F  4Y61B                178    0.196  39.257  -1.510   0.257   0.016   0.727
E R  4Y61B                179    0.607 139.095   0.316   0.115   0.016   0.868
B F  4Y61B                180    0.160  32.052  -0.437   0.058   0.017   0.925
E V  4Y61B                181    0.513  78.863  -0.226   0.053   0.043   0.903
E L  4Y61B                182    0.455  83.311   0.868   0.018   0.088   0.893
B L  4Y61B                183    0.073  13.458  -0.159   0.020   0.205   0.775
E T  4Y61B                184    0.341  47.241   0.603   0.022   0.359   0.619
E H  4Y61B                185    0.407  73.997   1.008   0.018   0.846   0.136
B L  4Y61B                186    0.061  11.169   0.588   0.018   0.846   0.136
E D  4Y61B                187    0.282  40.622   0.083   0.021   0.756   0.223
B L  4Y61B                188    0.083  15.289  -0.747   0.020   0.205   0.775
E R  4Y61B                189    0.494 113.080  -0.760   0.018   0.047   0.935
E G  4Y61B                190    0.556  43.773  -0.490   0.018   0.047   0.935
B N  4Y61B                191    0.164  24.024  -1.149   0.018   0.088   0.893
E R  4Y61B                192    0.433  99.065   0.428   0.022   0.359   0.619
B L  4Y61B                193    0.121  22.100  -1.426   0.022   0.552   0.426
E K  4Y61B                194    0.442  90.878   0.538   0.022   0.552   0.426
E V  4Y61B                195    0.499  76.758   0.059   0.004   0.420   0.576
B M  4Y61B                196    0.100  20.090   1.023   0.005   0.262   0.733
E P  4Y61B                197    0.408  57.966  -0.016   0.004   0.085   0.910
E F  4Y61B                198    0.447  89.773  -0.408   0.181   0.016   0.803
E A  4Y61B                199    0.717  78.991  -1.142   0.321   0.003   0.675
E G  4Y61B                200    0.456  35.864  -1.241   0.321   0.003   0.675
B V  4Y61B                201    0.194  29.864  -1.101   0.339   0.016   0.645
B L  4Y61B                202    0.178  32.610  -1.423   0.257   0.016   0.727
E E  4Y61B                203    0.636 111.179  -0.311   0.115   0.016   0.868
E H  4Y61B                204    0.541  98.499  -0.233   0.053   0.005   0.942
B I  4Y61B                205    0.074  13.635  -0.185   0.018   0.019   0.964
E G  4Y61B                206    0.548  43.128   0.131   0.018   0.019   0.964
E G  4Y61B                207    0.414  32.566   1.170   0.018   0.088   0.893
B I  4Y61B                208    0.059  11.007   0.486   0.004   0.197   0.799
E M  4Y61B                209    0.363  72.576   0.989   0.004   0.420   0.576
E E  4Y61B                210    0.410  71.540   1.455   0.001   0.900   0.099
B I  4Y61B                211    0.035   6.456   0.546   0.001   0.900   0.099
E Q  4Y61B                212    0.282  50.419   0.966   0.011   0.918   0.071
B L  4Y61B                213    0.035   6.482  -0.718   0.004   0.514   0.481
E E  4Y61B                214    0.395  69.041  -0.307   0.004   0.138   0.858
E E  4Y61B                215    0.656 114.533  -0.640   0.005   0.015   0.979
B N  4Y61B                216    0.092  13.454  -0.001   0.005   0.015   0.979
E P  4Y61B                217    0.370  52.475   0.423   0.019   0.141   0.840
B W  4Y61B                218    0.082  19.769  -0.587   0.022   0.359   0.619
E N  4Y61B                219    0.437  64.006   0.621   0.004   0.420   0.576
B C  4Y61B                220    0.076  10.670  -0.505   0.022   0.359   0.619
B T  4Y61B                221    0.257  35.646  -0.339   0.018   0.088   0.893
B C  4Y61B                222    0.098  13.703   0.073   0.181   0.016   0.803
E D  4Y61B                223    0.501  72.136   0.960   0.339   0.016   0.645
B L  4Y61B                224    0.077  14.172  -1.123   0.622   0.015   0.363
E L  4Y61B                225    0.310  56.779   0.446   0.879   0.010   0.111
E P  4Y61B                226    0.341  48.388  -0.574   0.858   0.002   0.139
B L  4Y61B                227    0.064  11.737  -0.636   0.923   0.002   0.076
B K  4Y61B                228    0.187  38.445   0.384   0.923   0.002   0.076
E A  4Y61B                229    0.499  54.990   1.325   0.970   0.001   0.030
B W  4Y61B                230    0.148  35.546   0.653   0.970   0.001   0.030
B L  4Y61B                231    0.041   7.599  -1.132   0.923   0.002   0.076
E D  4Y61B                232    0.489  70.407   0.988   0.923   0.002   0.076
E T  4Y61B                233    0.588  81.528   0.801   0.858   0.002   0.139
B I  4Y61B                234    0.153  28.286   0.584   0.522   0.016   0.462
E T  4Y61B                235    0.515  71.500  -0.635   0.181   0.016   0.803
B V  4Y61B                236    0.235  36.089  -1.380   0.058   0.017   0.925
E F  4Y61B                237    0.520 104.324  -0.697   0.053   0.043   0.903
E V  4Y61B                238    0.392  60.189  -1.262   0.053   0.043   0.903
E G  4Y61B                239    0.564  44.347  -1.066   0.018   0.019   0.964
E E  4Y61B                240    0.489  85.498   0.194   0.052   0.084   0.864
B I  4Y61B                241    0.073  13.486  -0.419   0.021   0.451   0.528
E V  4Y61B                242    0.373  57.315   0.949   0.021   0.756   0.223
B C  4Y61B                243    0.100  14.082  -0.690   0.004   0.616   0.381
E E  4Y61B                244    0.333  58.175  -0.762   0.005   0.262   0.733
E T  4Y61B                245    0.381  52.831  -0.598   0.005   0.045   0.951
B P  4Y61B                246    0.184  26.124  -0.038   0.339   0.016   0.645
E F  4Y61B                247    0.614 123.270  -0.122   0.502   0.002   0.495
E R  4Y61B                248    0.539 123.431   0.318   0.522   0.016   0.462
B L  4Y61B                249    0.135  24.719  -0.431   0.522   0.016   0.462
E H  4Y61B                250    0.542  98.626   0.274   0.339   0.016   0.645
E G  4Y61B                251    0.870  68.438  -0.152   0.058   0.017   0.925
E K  4Y61B                252    0.272  55.971   2.037   0.018   0.088   0.893
E D  4Y61B                253    0.436  62.871   1.805   0.020   0.205   0.775
B V  4Y61B                254    0.047   7.224  -0.159   0.066   0.296   0.638
E T  4Y61B                255    0.440  60.986   1.189   0.066   0.296   0.638
E Q  4Y61B                256    0.566 101.034   1.672   0.066   0.296   0.638
B L  4Y61B                257    0.049   8.917   1.058   0.056   0.142   0.802
E T  4Y61B                258    0.466  64.606   1.797   0.018   0.019   0.964
E R  4Y61B                259    0.543 124.347   0.798   0.115   0.016   0.868
E Q  4Y61B                260    0.733 130.860   0.548   0.058   0.017   0.925
E D  4Y61B                261    0.556  80.076   0.878   0.058   0.017   0.925
B L  4Y61B                262    0.071  13.018  -1.410   0.052   0.084   0.864
E C  4Y61B                263    0.336  47.202   0.103   0.019   0.141   0.840
B P  4Y61B                264    0.184  26.124  -0.996   0.019   0.141   0.840
E R  4Y61B                265    0.535 122.607   0.434   0.018   0.019   0.964
E K  4Y61B                266    0.503 103.426  -0.387   0.003   0.003   0.994
E H  4Y61B                267    0.560 101.937  -0.719   0.003   0.003   0.994
E H  4Y61B                268    0.631 114.724  -0.605   0.005   0.015   0.979
E H  4Y61B                269    0.323  58.808  -1.220   0.005   0.045   0.951
E H  4Y61B                270    0.399  72.487  -0.951   0.005   0.015   0.979
E H  4Y61B                271    0.540  98.317  -0.130   0.005   0.015   0.979
E H  4Y61B                272    0.677 123.164  -1.983   0.003   0.003   0.994

Explain the output.  Go back.
```

---
